# Editorial: Advanced technologies of UAV application in crop pest, disease and weed control

**DOI:** 10.3389/fpls.2023.1253841

**Published:** 2023-08-16

**Authors:** Ruirui Zhang, Andrew Hewitt, Longlong Li, Huizhu Yuan, J. Connor Ferguson, Liping Chen

**Affiliations:** ^1^ National Research Center of Intelligent Equipment for Agriculture, Beijing Academy of Agricultural and Forestry Sciences, Beijing, China; ^2^ National Center for International Research on Agricultural Aerial Application Technology, Beijing, China; ^3^ Centre for Pesticide Application and Safety, The University of Queensland, Brisbane, QLD, Australia; ^4^ State Key Laboratory for Biology of Plant Diseases and Insect Pests, Institute of Plant Protection, Chinese Academy of Agricultural Sciences (CAAS), Beijing, China; ^5^ Sesaco Corporation, Austin, TX, United States

**Keywords:** UAV (unmanned aerial vehicle), plant protection, aerial spray application, remote sensing, chemical application

In recent years, the use of unmanned aerial vehicles (UAVs) as auto-spraying machines for plant protection has been increasing (Hu et al., 2022). Recent research has been conducted on the spray deposition/drift patterns of plant protection UAVs (Tang et al., 2020; Li et al., 2022), but further exploration is required to ensure their efficient and accurate application. This Research Topic aims to conduct in-depth studies on new technologies for the application of plant protection UAVs in crop pest, disease, and weed control. The published articles cover four topics including pest, disease, weed detection, and identification; canopy remote sensing and identification; strategies for improving the spray quality of UAV applications; and spray drift assessment. This research aims to serve as a reference for new theories and advanced technologies and to optimize the use of UAVs in crop pest, disease, and weed control, helping to expand the application potential of plant-protection UAVs.

## Pest, disease, and weed detection and identification

Accurate target detection is crucial for establishing prescriptions for chemical applications and enabling variable spraying with UAVs. This has become even more important in the application of high-speed plant-protection UAVs, where there is increased demand for precise target identification.

Xia et al. presented a method for identifying resistant weed biotypes using multispectral and RGB images based on a deep convolutional neural network (DCNN). They developed a weed spectral resistance index (WSRI) that compared susceptible and resistant weed biotypes. By fusing multispectral and RGB images, they enhanced the accuracy of resistance identification. The DCNN achieved impressive field accuracies of 81.1% and 92.4% for barnyard grass and velvet leaves, respectively.

In another study, Yu et al. developed a weed vegetation index (WDVI_NIR_) by utilizing the reflectance of three bands—red, green, and near-infrared— captured by multispectral images. Compared with the traditional vegetation indices of NDVI, LCI, NDRE, and OSAVI, WDVI_NIR_ showed the most effective ability to identify weeds from rice, water cotton, and soil, with a weed identification accuracy of 93.47% and a kappa coefficient of 0.859.

In addition to weed identification, Lu et al. proposed a method for estimating leaf chlorophyll content in jujube leaves infested by leaf mites using soil plant analysis development (SPAD). Their approach aimed to estimate the severity of mite infestation by correlating it with the SPAD values of jujube leaves. A particle swarm optimization-extreme learning machine (PSO-ELM) for SPAD and vegetation indices were established and exhibited superior accuracy (*R* ^2^ = 0.856, *RMSE* = 0.796) when compared with the ELM model alone (*R* ^2^ = 0.748, *RMSE* = 1.689). This indirect measurement approach is a novel method for detecting and identifying pests and diseases.

## Canopy remote sensing and identification

A high-precision canopy segmentation methodology called MPAPR R-CNN, specifically designed for high-density cultivation orchards, was proposed utilizing low-altitude visible light images (Zhang et al.). This method accurately identifies and segments the canopy edge, which can be affected by tree branch extensions and shadow obstructions. The researchers employed a Mask R-CNN as the base segmentation algorithm, incorporating a path augmentation feature pyramid network (PAFPN) and the PointRend algorithm to achieve precise boundary delineation of apple tree canopies. Training with the PAFPN and Point-Rend backbone head resulted in significant improvements, with average precision scores increasing by 8.96%.

Li et al. introduced a deep-learning-based method for counting maize plants using image datasets. A real-time detection model for maize plants was trained based on YOLOv5, and a tracking and counting approach was developed using Hungarian matching and Kalman filtering algorithms. The maize plant counts using this method exhibited a high correlation with the manual count results (*R* ^2 =^ 0.92). In a separate study, Zhang et al. proposed an improved lightweight network, improved YOLOv5s, for dragon fruit detection in an all-weather environment. The results demonstrated that the model achieved a mean average precision (mAP) of 97.4%, precision (*P*) of 96.4%, and recall rate (*R*) of 95.2%. Compared with the original YOLOv5s network, the improved model exhibited a reduction in model size, params, and floating-point operations (FLOPs) by 20.6%, 18.75%, and 27.8%, respectively.

## Strategies for improving spray quality of UAV application

Liu et al. conducted a study that investigated the impact of adjuvants on the physicochemical properties of defoliant solutions and droplet deposition in defoliation spraying using plant-protection UAVs. They aimed to determine the type of adjuvant that enhances the effect of defoliation on pepper plants. Previous research has demonstrated that the appropriate addition of additives to a spray solution can reduce spray drift and improve droplet adhesion to leaves. By employing this method, droplet deposition increases, and the defoliation effect is achieved. Among the adjuvants used in their study, Puliwang was the most efficient for the aerial application of defoliants.

Downwash airflow is a prominent characteristic of plant-protection UAV operations. Chang et al. employed the Lattice Boltzmann Method (LBM) to investigate the rotor flow field of a quadrotor plant-protection UAV at different speeds. As the rotor speed increased, the maximum velocity and vorticity of the wind field under the rotor increased gradually, whereas the ultimate values of the velocity and vorticity decreased owing to the emergence of turbulence. This is expected to reveal and comprehend the changes in the rotor flow field of plant-protection UAVs as the pesticide loading dynamically evolves.

Considering the limited deposition in the lower canopy when using plant-protection UAVs, particularly in high-density fruit trees, Jiang et al. developed a stereoscopic plant-protection system (SPS) consisting of a small swing-arm ground sprayer and a UAV sprayer. This approach demonstrated that the density of vertical droplet deposition in the canopies ranged from 90 to 107 deposits/cm^2^, and the uniformity was 38.3% higher than that of conventional methods.

## Spray drift assessment

The primary current challenge to the widespread adoption of plant-protection UAVs is the potential risk associated with spray drift exposure in pesticide applications. Accurate measurement of spray drift is crucial because it serves as the basis for scientifically developing spray technology and selecting appropriate operating environments. Li et al. presented a method for evaluating spray drift based on 3D point cloud data from a light detection and range technique (LiDAR). LiDAR measurements provide valuable spatial information, including the height and width of drifting droplets (Liu et al., 2022). However, it is important to note that LiDAR detection is sensitive to droplet density or drift mass in space, and drift clouds with lower densities and smaller droplet sizes may not be effectively detected by LiDAR. This method has the potential to serve as an alternative tool for evaluating the drifts of different spray configurations, although it may not provide direct measurements of the actual spray drift mass.

## Conclusion

Plant-protection UAVs are a promising tool, having shown significant success in East Asia, particularly in China, which is the focus of the articles in this Research Topic. All of these published manuscripts were funded by the Chinese government. Australian scholars have also contributed to the study of spray drift evaluation using 3D LiDAR. The greatest challenges faced by plant-protection UAVs in global applications are safety concerns and incidents of environmental pollution caused by the off-target drift of high-concentration pesticides induced by downwash flow at a higher operating altitude. In addition, some users have a limited understanding of plant-protection UAVs, particularly regarding the feasibility of using a minimal application volume rate for pest and disease control. Nevertheless, the situation may eventually change with new technological developments, given the exceptional operational capabilities of plant-protection UAVs in China.

We hope that the readers will find this Research Topic a valuable reference for understanding state-of-the-art advanced technologies in UAV chemical applications and their practical implications for precise spraying.

## Author contributions

RZ: Conceptualization, Funding acquisition, Investigation, Project administration, Writing – original draft, Writing – review & editing. AH: Validation, Writing – review & editing. LL: Data curation, Writing – original draft, Writing – review & editing. HY: Validation, Writing – review & editing. JF: Validation, Writing – review & editing. LC: Validation, Writing – review & editing.
